# Meeting Abstracts from the 5^th^ B Chromosome Conference

**DOI:** 10.1186/s12919-023-00284-9

**Published:** 2023-11-30

## I-1 Welcome to the 5^th^ B Chromosome Conference

### Jelena Blagojević

#### Institute for Biological Research „Siniša Stanković“   – National Institute for the Republic of Serbia, University of Belgrade, Belgrade, Serbia

##### **Correspondence:** Jelena Blagojević (jelena.blagojevic@ibiss.bg.ac.rs)

*BMC Proceedings 2023*, **17(20)**:I-1

The discovery of B chromosomes (Bs), accessory or additional chromosomes, occurred very early in the history of cytogenetics. Edmund Wilson (1907), working on hemipteran chromosomes, observed structures that appeared to be additional to the main karyotype. These structures were present in only a fraction of individuals. Eleven years later, in 1928, the term 'B chromosome' was officially introduced. Lowell Fitz Randolph, who studied variations in maize chromosomes, proposed that stable chromosomes of the standard complement be called 'A chromosomes' and those that occur in addition to the standard complement and are variable in number and morphology be called 'B chromosomes' (Randolph 1928). Since then, numerous publications have permanently widened knowledge of these additional chromosomes. Bs feature in populations of numerous species in almost all major phylogenetic groups. They are most common (D'Ambrosio *et al.* 2017) and best studied in plant species since they are present in the genomes of agriculturally important crops, namely maize and rye. In 1981. Jones and Rees published their book “B-Chromosomes," in which the authors united and systematized research in the field for the first time. This book become a primer for all B researchers. In the following years, new methods and technological developments made research much more exciting and diverse. Thus began the history of conferences dedicated to B chromosomes.

The inaugural B Chromosome Conference (BCC) was held in Miraflores de la Sierra, Madrid, Spain, from 21 to 25 September 1993. This important event encompassed five sessions dedicated to B chromosome research. These sessions covered Polymorphisms and geographical distribution, Transmission: non-Mendelian heredity, Genetic structure and organization, Phenotypic effects, and Population dynamics. An impressive international group of researchers from 12 countries actively participated in the conference and 48 studies were presented. At the BCC, a groundbreaking proposal redefined B chromosomes as "dispensable supernumerary chromosomes that do not recombine with A chromosomes and follow their own evolutionary pathway" (Beukeboom, 1994). This description of the B chromosome has remained unchanged, and most researchers agree that it describes the properties of all B chromosomes regardless of species. The 2nd BCC was held in Granada, Spain, from 26 to 29 June 2004. Participants from 15 countries explored different aspects of B chromosomes in five sessions, focusing on Frequency and meiotic behaviour, Transmission, Phenotypic effects, DNA composition and origin, and Evolution. New molecular cytogenetic techniques dominated in conference publications, opening up new possibilities for comparative analyses and enabling the mapping of DNA sequences within chromosomes. A decade later, the 3rd B-Chromosome Conference was held in Gatersleben, Germany, from 7 to 9 April 2014. Participants from 11 countries gathered to address the latest advances in B chromosome research. The conference featured discussions on five key areas, including the Structure and evolution of animal and plant B chromosomes, B chromosome effects and genes, Population genetics of B chromosomes, Segregation behaviour and drive of B chromosomes, and Novel analysis methods and applications of B chromosomes. A particular highlight of this conference was the exploration of large-scale DNA and RNA analyses, taking B chromosome biology to a new level. Finally, the 4th B-Chromosome Conference was held in Botucatu, Brazil, from 20-23 July 2019. This conference had 34 talks and poster presentations spanning diverse subjects such as Education in chromosome Science, Structure, composition, and evolution of B chromosomes, Genes, and B chromosome effects, Population genetics of B chromosomes, Segregation behaviour and drive of B chromosomes, and New technologies and applications of B chromosomes. The main impression from this conference was the significant influence of developments in methods and tools in genomics, bioinformatics, as well as functional protein analyses, all of which are driving the Omics phase in the study of B chromosomes, termed “B-omics” (Ahmad & Martins, 2019).

During preparations for the 5th B-Chromosome Conference (5BCC), rapid and significant progress was observed in the germ-restricted chromosomes (GRCs) study. GRCs are additional chromosomes found only in germ cells and are eliminated from somatic cells in many avian and dipteran species. Despite their specificity, GRCs share many features of B chromosomes. In view of this intriguing connection, we have decided to extend a special invitation to researchers dedicated to this fascinating topic and to encourage them to participate in the 5BCC.

The 5th B Chromosome Conference will be held in Petnica, Serbia, from 14^th^ to 17^th^ October 2023. The conference will take place in five sessions: Constitution, gene composition, Dynamics in populations, B chromosome effects, Segregation behaviour, Origin and evolution. The conference will unite the most prominent chromosome biologists for a stimulating exchange of ideas and insights. We hope that the conference will help establish better collaboration and enable progress in B chromosome research.

**References**

1. Wilson, E.B. The Supernumerary Chromosomes of Hemiptera. Sci. New Ser. 1907; 26:870–871.
2. Randolph, L.F. Types of Supernumerary Chromosomes in maize. Anat. Rec. 1928; 41:102.
3. D'Ambrosio U, Alonso-Lifante MP, Barros K, Kovařík A, de Xaxars GM, Garcia S. B-chrom: a database on B-chromosomes of plants, animals and fungi. New Phytol. 2017; 201, 3:635-642.
4. Jones RN, Rees H. B Chromosomes. London: Academic Press;1981.
5. Beukeboom L. Bewildering Bs: an impression of the 1st B-Chromosome Conference. Heredity 1994; 73:328–336.
6. Ahmad SF, Martins C. The Modern View of B Chromosomes Under the Impact of High Scale Omics Analyses. Cells 2019; 8:156.

## S0 Key note talk - B chromosome research – *Quo vadis*?

### Andreas Houben

#### Leibniz Institute of Plant Genetics and Crop Plant Research (IPK), Gatersleben, Seeland, Germany

##### **Correspondence:** Andreas Houben (houben@ipk-gatersleben.de)

*BMC Proceedings* 2023, **17(20)**:S0

**Background**

In the field of B chromosome biology, significant progress has been made based on the development and application of new technologies. I will discuss how methods like whole genome sequencing, RNA-seq, and others were used to address fundamental B chromosome research questions.

## Session 1 - Constitution, gene composition of the B chromosomes

### S1-O1 Diversity of the maize B chromosome and its gene pool

#### Jan Bartoš

##### Centre of Plant Structural and Functional Genomics, Institute of Experimental Botany of the Czech Academy of Sciences, Olomouc, Czech Republic

###### **Correspondence:** Jan Bartoš (bartos@ueb.cas.cz)

*BMC Proceedings* 2023, **17(20)**:S1-O1

The maize B chromosome is one of the best studied supernumerary chromosomes in the plant kingdom. It has a unique accumulation mechanism based on non-disjunction at the second pollen mitosis, coupled with preferential fertilisation of the egg by B-containing sperm. To date, deficiency mapping uncovered several loci involved in these processes, but no particular genes driving the mechanisms of accumulation have been identified so far. Analysis of the diversity at the level of individual genes will point to conserved ones, which should comprise relevant candidates.

The presence of the B chromosome in various maize landraces has been documented. We screened about 800 accessions from the CIMMYT maize collection and found the B chromosome in more than 300 of them. We then selected one hundred accessions (preferably with two B chromosomes) representing the entire geographical and ecological distribution of maize and sequenced their DNA. Using the improved reference sequence of the B chromosome, over 800 thousand high quality SNPs were identified among the accessions studied. These SNPs are now being used to study diversity at the level of individual protein-coding genes, with the aim of determining genes under positive selection.

**Acknowledgements**

The work was supported by the Ministry of Education, Youth and Sports (award no. LTT19007) and the Czech Science Foundation (award no. 23-04887S).

### S1-O2 The microchromosome of the Amazon Molly: a nascent B chromosome?

#### Irene da Cruz^1^, Dunja K. Lamatsch^2^, Edward Rice^3^, Indrajit Nanda^4^, Vladimir A. Trifonov^2^, Wesley Warren^3^, Manfred Schartl^1,5^

##### ^1^Developmental Biochemistry, Biozentrum, University of Würzburg, Würzburg, Germany; ^2^Research Department for Limnology, University of Innsbruck,Mondsee, Austria; ^3^Department of Animal Sciences, University of Missouri, Bond Life Sciences Center, Columbia, USA; ^4^Human Genetics, Biozentrum, University of Würzburg, Würzburg, Germany; ^5^The *Xiphophorus* Genetic Stock Center, Texas State University, San Marcos, USA

###### **Correspondence:** Dunja K. Lamatsch (Dunja.Lamatsch@uibk.ac.at)

*BMC Proceedings* 2023, **17(20)**:S1-O2

**Background**

The Amazon molly, *Poecilia formosa* is a clonal species of livebearing fish that reproduces by sperm-dependent parthenogenesis. Diploid oocytes are generated by apomixis due to a failure in the synapsis of homologous chromosomes. The development of embryos from the unreduced eggs is then triggered by insemination from males of sympatric “host” species. Karyogamy does not happen and the paternal DNA is usually degraded, while the maternal nucleus starts dividing. The commonly accepted prediction for organisms that have no meiotic recombination are a low genetic diversity and decay of genes, which both should lead to rapid extinction. However, *P. formosa* has a much higher evolutionary age than calculations predicted, and is a very successful colonizer in north-eastern Mexico. It has been hypothesized that rare events of introgression of fresh genetic material of paternal origin can counteract the negative effects of accumulation of deleterious mutations and low genetic diversity. Such introgression events manifest as host species derived microchromosomes if a small fraction of the parental genome is included. This happens, when the paternal host sperm DNA is not completely excluded after insemination of the diploid egg.

**Materials and Methods**

We use cytogenetics, whole genome sequencing, transcriptomics and population genomics to characterize microchromosomes and to elucidate their possible biological function and evolutionary meaning.

**Results**

Microchromosomes are found in about 25% of Amazon mollies. All microchromosomes that are transmitted to the clonal offspring have a centromere, where those which also have a telomere show a higher heritability and somatic stability. Clonal transmission ranges from 40 to 99%.

Haplotype-resolved chromosome level genomes of fish with and without microchromosomes were established and the entire microchromosome was assembled in a 5 Mb scaffold. It is derived from a proximal fragment of chromosome 1 of the sperm donor species with a high repeat and transposable element content, which make up more than 50% of the sequence. It contains 85 protein coding genes, many of which contribute to the transcriptome of brain, liver, and skin.

**Conclusions**

Microchromosomes of *P. formosa* form a relatively stable component of the “active” genome. Being derived from a recombining host species genome they increase the genetic diversity and may compensate for corrupted orthologous sequences in the ancient A-chromosome complement. Being only 35 years old, the fully sequenced Amazon molly microchromosome can inform about the origin and early evolutionary steps of B-chromosomes.

### S1-O3 Contributions of Accessory (B) chromosomes to the cross-kingdom fungal pathogenesis of *Fusarium oxysporum*

#### Li-Jun Ma

##### Department of Biochemistry and Molecular Biology, University of Massachusetts Amherst, Amherst, USA

###### **Correspondence:** Li-Jun Ma (lijun@umass.edu)

*BMC Proceedings* 2023, **17(20)**:S1-O3

*Fusarium oxysporum* is a cross-kingdom fungal pathogen that infects both plants and animals. Long regarded as a highly destructive plant pathogen, *F. oxysporum* was recently recognized by WHO as a high-priority threat to human health. An *F. oxysporum* genome is divided into two compartments: core chromosomes (CCs) and accessory chromosomes (ACs). CCs are conserved, vertically transmitted from parent to offspring, and involved in essential housekeeping functions, whereas lineage- or strain-specific ACs, also known as B chromosomes, are not conserved, are thought to be horizontally transmitted, and are associated with specialized functions. My lab was the first to discover ACs in a tomato wilt-causing *F. oxysporum* strain and subsequently reported distinct sets of ACs in two human pathogenic strains. Our research documented that horizontally transmitted ACs of *F. oxysporum* genomes determine host-specific virulence and hold the key to a fundamental question of why a single species possesses a rare capacity to overwhelm the host’s sophisticated immune system and cause serious disease in both plants and animals. Focusing on fungal–host interfaces, we have established *F. oxysporum* infection models using mouse as an animal host and *Arabidopsis* and tomato as plant hosts. Drawing on our expertise in bioinformatics, molecular characterization, forward and reverse genetics, and short-term experimental evolution, I will report our current understanding on how ACs contribute to host-specific virulence. I will report our efforts in identifying master regulators used by both plant and human pathogens to coordinate the functions of ACs and the core genome. To dissect host defence, we have employed single-nucleus RNA sequencing using the *Fusarium*–*Arabidopsis* interaction model. Ultimately, we aim to apply the gained knowledge to control this threatening cross-kingdom pathogen.

### S1-O4 Searching for B chromosomes in high contiguity genome assemblies across Tree of Life

#### Thomas Mathers, Alex Makunin, Kamil Jaron

##### Tree of Life, Wellcome Sanger Institute, Hinxton, Cambridgeshire, UK

###### **Correspondence:** Alex Makunin (am60@sanger.ac.uk)

*BMC Proceedings* 2023, **17(20)**:S1-O4

**Background**

B chromosomes were recorded in various eukaryotic taxa for over a century, predominantly using cytogenetics methods. For a smaller subset of model species, more detailed studies were performed to reveal variability in B chromosome mitotic and meiotic behaviour and/or resulting patterns of their population, germline and somatic frequencies, as well as their morphological heterogeneity. Patterns of Bs genetic content were historically revealed mostly for repetitive sequence, and with the onset of high throughput short read sequencing, surveys of the non-repetitive genetic content became possible. As a result, B chromosomes turned out to be complex mixtures of multiple regions homologous with the core genome, but their high-level structure often remained unknown.

**Materials and Methods**

A great opportunity to discover and characterise novel or well-known B chromosomes is within the large sequencing consortia generating chromosome-level assemblies with minimal number of gaps by relying on the combination of long reads and Hi-C data.

**Results**

Within the first thousand genomes of the Darwin Tree of Life, we have accidentally stumbled upon at least two species showing signatures of B chromosomes - the field maple, *Acer campestre*, and the Ingrailed Clay, *Diarsia mendica*. The two B chromosomes were both assembled in a single scaffold, demonstrating a high potential for this approach.

**Conclusions**

To further progress our ability to recognise and characterise B chromosomes in biodiversity sequencing efforts, we propose assembling a panel of well-characterised species with B chromosomes and sequence them telomere-to-telomere via trio method. We argue that generation of gapless haplotype-resolved assemblies with known B chromosome status in a set of model species would allow not only for deeper insights into non-canonical chromosome biology, but also for generation of a benchmarking dataset for robust detection of B chromosomes in long-read assemblies, which would inform further cytogenetic, molecular, or population genetics research focused on Bs in new species.

**Acknowledgements**

This work was supported by Wellcome through core funding to the Wellcome Sanger Institute (206194) and the Darwin Tree of Life Discretionary Award (218328).

### S1-O5 Sex determination in the Pachon cavefish, *Astyanax mexicanus*, by B-chromosomes

#### Manfred Schartl^1,2^, Boudjema Imarazene^3^, Amaury Herpin^3^, Alexandr Sember^4^, Sylvie Rétaux^5^, Sylvain Bertho^6^, Nicolas Rohner^6^, Yann Guiguen^3^

##### ^1^Developmental Biochemistry, Theodor-Boveri Institute, Biocenter, University of Würzburg, Germany and; ^2^The Xiphophorus Genetic Stock Center, Department of Chemistry and Biochemistry, Texas State University, San Marcos, Texas, USA; ^3^INRAE, LPGP, Rennes, France; ^4^Laboratory of Fish Genetics, Institute of Animal Physiology and Genetics, Czech Academy of Sciences, Czech Republic; ^5^Université Paris-Saclay, CNRS, Institut des Neurosciences Paris-Saclay, Gif sur Yvette, France; ^6^Stowers Institute for Medical Research, Kansas City, Missouri, USA

###### **Correspondence:** Manfred Schartl (phch1@biozentrum.uni-wuerzburg.de)

*BMC Proceedings* 2023, **17(20)**:S1-O5

**Background**

Sex chromosomes generally evolve from a classical type-A chromosome complement, and relatively few alternative models have been proposed so far. B-chromosomes (Bs) are supernumerary chromosomes that are found in all major lineages of plants and animals, and they are often considered as selfish genetic elements that behave as genome parasites. In vertebrates, the relationships between Bs, sex determination and sex chromosomes have often been suggested, but no functional evidence has been provided besides the frequent and mostly cytogenetic evidence of an association of Bs with sex.

**Results**

We previously showed that in the Pachón cavefish, *Astyanax mexicanus*, Bs occur almost exclusively in males (Imarazene *et al*., 2021). Based on a high-quality genome assembly of a B-carrying male, we characterized the Pachón cavefish B-chromosome sequence and found that it contains two duplicated loci of a putative Master Sex Determining (MSD) gene, namely growth differentiation factor 6b (gdf6b). Supporting its role as an MSD gene, we found that the Pachón cavefish gdf6b is expressed specifically in differentiating male gonads, and that its knockout induces male-to-female sex reversal in B-carrying males. This demonstrates that gdf6b is necessary for triggering male sex determination in Pachón cavefish. Altogether these results provide multiple and independent lines of evidence supporting the conclusion that the Pachón cavefish B is a bona-fide “B-sex” chromosome that can trigger male sex determination in this species. Further exploration of the association of this B chromosome with sex in additional populations of *Astyanax mexicanus* revealed a strong conservation of the “B-sex” chromosome system in many cave and surface populations of this species. However, another independently derived cavefish population, originating from the Molino cave, was characterized by a complete absence of B chromosomes. Based on a combination of population genomics and reference whole-genome sequencing of different individuals from different cavefish populations, we found that the Molino population has evolved an XX/XY sex chromosome system from classical A-type autosomes. Interestingly, a similar duplication of the gdf6b MSD gene as found in the Pachón cavefish was revealed on the Y chromosome of the Molino population, suggesting that this gene could also serve as MSD gene in the Molino population.

**Conclusions**

Two sex chromosome systems, an A-type (Molino) and B-type (Pachón) exist in different cavefish populations. Current genome comparisons suggest that the B-sex chromosome evolved secondarily from an ancestral Y-specific locus that is still preserved in the Molino cavefish population.

**Acknowledgements**

Funding was provided by the French National Research Agency (ANR) and the German Research Foundation (DFG)

**Reference**

1. Imarazene B, Du K, Beille S, *et al.* A supernumerary "B-sex" chromosome drives male sex determination in the Pachón cavefish, *Astyanax mexicanus*. Curr. Biol. 2021; 31(21):4800-4809.e9.

### S1-O6 Meiotic telomeres of vole’s B chromosomes: shelterin and telomere-supporting proteins provide a link to the nuclear membrane

#### Sergey N Matveevsky^1^, Tatyana V Petrova^2^, Svetlana V Pavlova^3^, Oxana L Kolomiets^1^

##### ^1^Vavilov Institute of General Genetics, RAS, Moscow, Russia; ^2^Zoological Institute, RAS, Saint-Petersburg, Russia; ^3^A.N. Severtsov Institute of Ecology and Evolution, RAS, Moscow, Russia

###### **Correspondence:** Sergey N Matveevsky (sergey8585@mail.ru)

*BMC Proceedings* 2023, **17(20)**:S1-O6

**Background**

The mammalian meiotic telomere is protected by a specialized hexa-protein complex called shelterin, consisting of telomeric double-stranded DNA-binding proteins TRF1 and TRF2, single-stranded DNA-binding protein POT1, and bridging factors RAP1, TIN2 and TPP1 (Myler *et al*., 2021). CDK2 kinase can be attributed to telomere-supporting proteins, as it regulates the dynamics of nuclear envelope (NE) proteins and telomere attachment in meiotic prophase I (Viera *et al*., 2015). Telomeres are connected to the NE. In mammals, the meiosis-specific linker of the nucleoskeleton and cytoskeleton (LINC) complex composed of two transmembrane proteins, KASH5 in the outer nuclear membrane and SUN1 in the inner nuclear membrane, provides the telomere-NE link (Chen *et al.*, 2021). All of these components have been discovered for homologues that form synaptonemal complexes (SCs), that is, for meio-chromosomes of A-set, but not for Bs.

Meio-Bs are typically univalent that tend to co-locate with sex chromosomes in the sex body and are susceptible to meiotic inactivation. Despite the fact that telomeric DNA has previously been identified in Bs, the protein components of telomeres, as well as interactions with the nuclear envelope, have not yet been investigated. Here, for the first time, we analyse the distribution of some proteins involved in the construction of meio-telomeres of supernumeraries and their relationship to the nuclear envelope.

**Materials and Methods**

Two males of narrow-headed voles (*Lasiopodomys gregalis*) carrying two and three Bs were investigated. An immunocytochemical method was used to identify the localization of proteins in vole spermatocytes: the SCs protein - SYCP3, RAP1 proteins as an indicator of shelterin, KASH5 as a marker of LINC, and CDK2 kinase.

**Results**

SYCP3 was distributed irregularly along meio-Bs. RAP1 and KASH5 were detected as dot-like signals in the meio-telomere regions of As and Bs, with the only difference being that KASH5 dots were located slightly more distally. CDK2 was localized in dots in telomeres of As and Bs, as well as in the recombination nodules within SCs of A-set. Thus, the dot immuno-signals of RAP1, KASH5, and CDK2 in meio-telomeres were identical in A- (including asynaptic X and Y) and B-chromosomes.

**Conclusions**

*L. gregalis* meio-Bs, although they do not have homologs, unlike As, are common chromosomes equipped with formed shelterins and supporting proteins. These components facilitate the telomere-NE connections, enabling the transmission of cytoskeletal forces to regulate telomere dynamics. These additional and non-permanent chromosomes are equipped with all the necessary components and protective mechanisms that ensure telomeric homeostasis and genomic stability. This probably once again emphasizes the nonrandom appearance of Bs in the genome of animals and plants.

**References**

1. Myler LR, Kinzig CG, Sasi NK, Zakusilo G, Cai SW, de Lange T. The evolution of metazoan shelterin. Genes Dev. 2021; 35(23-24):1625-1641.
2. Viera A, Alsheimer M, Gomez R, Berenguer I, Ortega S, Symonds CE, Santamaria D, Benavente R, Suja JA. CDK2 regulates nuclear envelope protein dynamics and telomere attachment in mouse meiotic prophase. J. Cell Sci. 2015; 128(1):88-99.
3. Chen Y, Wang Y, Chen J, Zuo W, Fan Y, Huang S, Liu Y, Chen G, Li Q, Li J, Wu J, Bian Q, Huang C, Lei M. The SUN1-SPDYA interaction plays an essential role in meiosis prophase I. Nat. Commun. 2021; 12(1):3176.

### S1-P1 Reference genome assembly and comparative transcriptome analysis of developing embryos of *Sorghum purpureosericeum* with and without B chromosomes

#### Tereza Bojdová^1^, Miroslava Karafiátová^1^, Lucie Hloušková^1^, Jan Bartoš^1^, Andreas Houben^2^, Johannes Thiel^2^

##### ^1^Institute of Experimental Botany of the Czech Academy of Sciences, Centre of the Region Haná for Biotechnological and Agricultural Research, Olomouc, Czech Republic; ^2^Leibniz Institute of Plant Genetics and Crop Plant Research (IPK), Seeland, Germany

###### **Correspondence:** Tereza Bojdová (tereza.bojdova@seznam.cz)

*BMC Proceedings* 2023, **17(20)**:S1-P1

The wild species *Sorghum purpureosericeum* occasionally contains supernumerary B chromosomes in addition to its standard chromosome set. B chromosomes are dispensable elements that provide no advantage for their host organisms. In *S. purpureosericeum*, these B chromosomes exhibit somatic instability and are eliminated from most of the tissues during early embryonic development. This study aims to assemble the first reference genome sequence of *S. purpureosericeum* and to identify B chromosome-contigs based on sequencing data of micro-dissected B chromosomes and flow-sorted B chromosome-containing micronuclei. To identify differentially expressed genes that might drive B chromosome elimination in *S. purpureosericeum* tissues, an extensive RNA-Seq analysis of embryonic tissues was performed. Comparative transcriptome analysis of embryos at various developmental stages was carried out together with RNA-Sew analysis of laser-microdissected (LM) embryonic regions supposed to actively undergo B chromosome elimination. Preliminary results show a significant group of upregulated genes in B+ samples and indicate a potential role of B chromosomes in gene expression regulation, mainly during early embryo development.

**Acknowledgements**

The work was supported by The Czech Science Foundation (grant no. 22-02108S) and by The Czech Academy of Sciences (project no. DAAD-22-02).

### S1-P2 Transcriptome atlas of the maize B chromosome

#### Lucie Hloušková^1,2^, Zuzana Tulpová^1^, Nicolas Blavet^1^, Radim Svačina^1^, Kateřina Holušová^1^, Miroslava Karafiátová^1^, Jan Bartoš^1^

##### ^1^Institute of Experimental Botany of the Czech Academy of Sciences, Centre of Plant Structural and Functional Genomics, Olomouc, Czech Republic; ^2^Department of Cell Biology and Genetics, Palacký University, Olomouc, Czech Republic

###### **Correspondence:** Lucie Hloušková (hlouskoval@ueb.cas.cz)

*BMC Proceedings* 2023, **17(20)**:S1-P2

Maize (*Zea mays* L.) is one of the most important crops. It serves as a well established model for biological research and the maize B chromosome has been studied for many decades. However, the gene expression of the maize B chromosome across different plant tissues has not been thoroughly described. Here we present the first results of a comprehensive gene expression analysis of 15 maize tissues. We identified B-chromosome specific genes expressed in various developmental stages and plant organs. Roughly, one third of B-chromosome-localized genes are expressed in at least one of the tissues. Further, the effect of the B chromosome on the expression of A-chromosomal complement was investigated. This effect is most pronounced in reproductive organs. The transcriptome analysis of developing pollen indicated candidates for key B-chromosome accumulation mechanism, non-disjunction in the second pollen mitosis.

**Acknowledgements**

The work was supported by the Ministry of Education, Youth and Sports (award no. LTT19007) and Czech Science Foundation (award no. 23-04887S).

## Session 2 - Dynamics of the B chromosomes in the population

### S2-PL1 B chromosomes in populations of *Apodemus flavicollis* – never ending story

#### Mladen Vujošević, Marija Rajičić, Ivana Budinski, Branka Bajić, Tanja Adnađević, Jelena Blagojević

##### Department of Genetic Research, Institute for Biological Research “Siniša Stanković” – National Institute of the Republic Serbia, University of Belgrade, Belgrade, Serbia

###### **Correspondence:** Mladen Vujošević (mladenvu@sbb.rs)

*BMC Proceedings* 2023, **17(20)**:S2-O1

B chromosomes (Bs) are uncommon in mammals, featuring less than 2% of species, but six out of 22 species possess them in the genus *Apodemus*. In Serbia, over 40 populations of yellow-necked mice (*Apodemus flavicollis*) with varying habitat quality were studied. The frequency of B carriers (range: 0.09 to 0.67) increased with elevation, correlated with sub-zero days, and inversely with average temperature. B carrier frequency remained stable over eight years despite population density fluctuations, while seasonal variations linked to population size were observed. Overcrowding stress reduced B carriers among pre-reproductive individuals but enhanced survival in suboptimal conditions.

Phenotypic traits and B frequency were correlated, influencing cranial morphometric development. B carriers showed distinct developmental pathways for cranial traits, suggesting environment-specific benefits. Bs' presence did not affect carrier fecundity or fertility and was evenly distributed across age groups. B chromosomes (up to five) displayed common structures across populations in Serbia and Eastern Europe, possibly originating from sex chromosome pericentromeric regions. B-specific chromatin spatially resembled pericentromeric sex chromosomes, suggesting a similar mechanism to bypass meiotic checkpoints.

Current data support a heterotic model in *A. flavicollis*. Bs likely contribute to species adaptability by increasing genetic variability, potentially expanding their distribution.

**Acknowledgements**

This research was funded by the Ministry of Education, Science and Technological Development of the Republic of Serbia, Grant No. 173003.

### S2-O1 Supernumerary chromosomes contribute to karyotypic diversity within cryptic species of the subgenus *Stenocranius* (Cricetidae, Rodentia)

#### Svetlana V Pavlova^1,5^, Svetlana A Romanenko^2^, Sergey N Matveevsky^3^, Aleksander N Kuksin^4^, Ivan A Dvoyashov^5^, Yulia M Kovalskaya^1^, Tatyana V Petrova^5^

##### ^1^A.N. Severtsov Institute of Ecology and Evolution, RAS, Moscow, Russia; ^2^Institute of Molecular and Cellular Biology, SB RAS, Novosibirsk, Russia; ^3^Vavilov Institute of General Genetics, RAS, Moscow, Russia; ^4^Tuvinian Institute for Exploration of Natural Resources, SB RAS, Kyzyl, Russia; ^5^Zoological Institute, RAS, Saint-Petersburg, Russia

###### **Correspondence:** Svetlana V Pavlova (svetpavlova@yandex.ru)

*BMC Proceedings* 2023, **17(20)**:S2-O2

**Background**

The subgenus *Stenocranius* includes two cryptic species, *Lasiopodomys raddei*, which occurs in South-Eastern Transbaikalia and widespread *L. gregalis*; the latter has three allopatric and genetically well-isolated lineages A, B and C having unclear taxonomic rank. Previous literature data published in the last century showed that most of the studied narrow-headed vole populations are characterised by a stable 2*n*=36, while in populations from Central Mongolia 2*n* has varied between 36 and 40 owing to the presence of one to four B chromosomes (Bs).

**Materials and Methods**

To identify speciation mechanisms within the subgenus *Stenocranius*, we analysed karyotypic variation of narrow-headed voles from previously unexplored regions of South Siberia, including the Altai-Sayan region and Transbaikal Region – the major centres of diversity within the subgenus. In total, 49 individuals from 15 new localities were karyotyped; a total sample of 121 individuals from 37 localities was analysed. To determine karyotypic differences we used both classic differential bandings as well as fluorescent *in situ* hybridisation with ribosomal and telomeric DNA probes. In addition, we examine the structure and meiotic silencing of Bs using immunocytochemical analysis of synaptonemal complexes (SCs) in *Stenocranius* pachytene spermatocytes.

**Results**

Two cryptic species differ in several chromosomal characteristics although initially they shared the same 2*n*=36. The Early Pleistocene relic *L. raddei* carries stable 2*n*=36 (FN*a*=52) without Bs; the G-banded karyotype is presented for the first time.

Intra-population karyotype variation of *L. gregalis* is mainly due to various number of Bs. Besides 2*n*=36 (FN*a*=50) karyotypes, the *L. gregalis* lineages carried 1-5 heterochromatic small acrocentric Bs. Karyotypes of the two species also differ in localisation of C-heterochromatic blocks and distribution patterns of rDNA in A chromosome sets and on Bs, whereas telomeric sequences show stable localisation across all examined karyotype variants.

Immunodetection of several meiotic proteins indicates that the meiotic Bs are transcriptionally inactive, located generally within the sex body and manifest a pattern of meiotic behaviour similar to that of sex chromosomes. These data allow us to suppose some homology of Bs to the sex chromosomes.

**Conclusions**

The increasing number of Bs can point to an evolutionary series from the older forms of *L. raddei* and *L. gregalis* lineage A (with almost stable 2*n*=36) to evolutionarily younger lineages B and C carrying one to five Bs, with complete loss of the initial chromosome set in lineage C.

**Acknowledgements**

This research was funded by the Russian Science Foundation (RSF) 22-24-00513.

### S2-O2 Variation in the frequency of B chromosomes in small isolated populations of *Apodemus flavicollis*

#### Jelena Blagojević^1^, Saša Malkov^2^, Marija Rajičić^1^, Aleksa Rončević^1^, Ivana Budinski^1^, Branka Bajić^1^, Milan Miljević^1^, Mladen Vujošević^1^

##### ^1^Department of Genetic Research, Institute for Biological Research “Siniša Stanković“ – National Institute of the Republic of Serbia, University of Belgrade, Belgrade, Serbia; ^2^Faculty of Mathematics, University of Belgrade, Belgrade, Serbia

###### **Correspondence:** Jelena Blagojević (jelena.blagojevic@ibiss.bg.ac.rs)

*BMC Proceedings* 2023, **17(20)**:S2-O3

**Background**

Long-term studies on B chromosome (Bs) dynamics in natural populations of yellow-necked mice, *Apodemus flavicollis,* have unveiled intriguing seasonal variations influenced by environmental factors. On average, one third of animals possessed Bs in large natural populations. Urbanization, a potent catalyst for genetic diversity alterations, brings about habitat fragmentation and degradation, producing patches of small forested areas within urban landscapes. These fragmented habitats pose significant challenges for small mammal populations as migration and gene flow are severely restricted. To comprehensively grasp the repercussions of urbanization on B chromosome frequency, we embarked on a study within the Belgrade region.

**Materials and Methods**

The frequency of individuals with B chromosomes in five urban isolated forests populations of *A. flavicollis* has been studied. Employing live animal traps, we collected samples, and chromosome preparation was done directly from the bone marrow. Each animal's karyotype was analysed through examination of twenty metaphase figures.

**Results**

A total of 246 animals were sampled across five forest sites within the Belgrade territory and underwent karyotyping. The prevalence of animals with Bs exhibited remarkable variability, ranging from 3% to 50%. Intriguingly, at four of the sites, the incidence of Bs was significantly lower (ranging from 3% to 13%) compared to the average frequencies observed in natural populations. Conversely, at one site, the frequency soared to an exceptionally high 50%. Since all studied localities are at short distances from each other, climatic factors could be considered as the same. However, the studied sites differ in size and the level of adaptation to people's urban life. In a bid to elucidate the driving factors behind frequency variation, we developed a model simulating the influence of genetic drift, the predominant evolutionary force in small, isolated populations.

**Conclusions**

Beyond investigations focused on the molecular structure and origin of B chromosomes, it becomes evident that a comprehensive understanding necessitates population-level research, shedding light on the intricate mechanisms governing their persistence across diverse environments.

**Acknowledgements**

This research was funded by the Ministry of Science, Technological Development and Innovation of the Republic of Serbia, Grants Nos. 451-03-9/2021-14/200007, 451-03-68/2022-14/200007 and 451-03-47/2023-01/200007.

### S2-P1 B chromosomes of the bat species *Nyctalus leisleri* from Serbia

#### Marija Rajičić^*^, Branka Bajić, Ivana Budinski, Milan Miljević, Aleksa Rončević, Jelena Blagojević

##### Department of Genetic Research, Institute for Biological Research “Siniša Stanković“ – National Institute of the Republic of Serbia, University of Belgrade, Belgrade, Serbia

###### **Correspondence:** Marija Rajičić (marija.rajicic@ibiss.bg.ac.rs)

*BMC Proceedings* 2023, **17(20)**:S2-P1

**Background**

B chromosomes (Bs) are infrequent in bat species, with only four known so far, including Lesser Noctule, *Nyctalus leisleri* (Kuhl, 1817). This western Palearctic species is widely distributed, though seldom captured, due to its late emergence from roosts or the fact they are tree dwellers, thus difficult to track. As a known long-distance migrant, it covers substantial distances between summer and winter roosts. Previously, a cytogenetical study investigated chromosome sets of four males from two Serbian locations, revealing karyotypes with two to five micro Bs. Notably, studies on female *N. leisleri* are scarce.

**Materials and Methods**

Recently, karyotypes and B's presence of additional specimens from southwestern Serbia (two males), and Belgrade (one female) were examined. Chromosome slides were made from the primary fibroblast cell cultures established from a piece of skin from the wing membrane. At least 20 metaphase plates per individual were assessed.

**Results**

The species’ standard chromosome complement counts 44 (42 autosomes and a sex chromosome pair), with extra chromosomes classified as Bs. In two males, counts were 2n=44+1B and 2n=44+2B, while the Belgrade female had 2n=44+2B. Notably, the X chromosome was a medium-sized metacentric and the Y chromosome was a small acrocentric, while all Bs were microchromosomes. A previous study from Poland from 1970 reported a female *N. leisleri* karyotype of 2n=46, but Bs were not mentioned due to the analysis of a single individual. Our findings of a female with 2n=44 and two additional Bs suggest that the Polish study likely shared the same scenario.

**Conclusions**

The paucity of B chromosomes in bats might be attributed to their small genome size, possibly linked to the energy demands of flight. Additionally, bats’ low reproductive rates could hinder Bs establishment and maintenance, as opposed to rodents, which exhibit the highest occurrence of Bs among mammals. This research marks the first confirmation of Bs in female *N. leisleri*, and supplements the limited understanding of additional chromosomes in bats with two more male karyotypes.

**Acknowledgements**

This work was supported by the Ministry of Science, Technological Development and Innovation of the Republic of Serbia (Grants No. 451-03-47/2023-01/200007).

### S2-P2 Different populations of *Apodemus peninsulae* in Siberia (Russia) show different degree of stability of Bs systems: results of 36 years of observations

#### Igor A Zhigarev^1^, Yuri Borisov^2^

##### ^1^Department of Zoology and Ecology, Institute of Biology and Chemistry, Moscow Pedagogical State University (MPSU), Moscow, Russia; ^2^Severtsov Institute of Ecology and Evolution, Russian Academy of Sciences, Moscow, Russia

###### **Correspondence:** Igor A Zhigarev (i.zhigarev@gmail.com)

*BMC Proceedings* 2023, **17(20)**:S2-P2

**Background**

Regular studies of abnormal karyotypes of *Apodemus peninsulae* in North Asia began in the 1970s. *A. peninsulae* revealed significant polymorphism in the number and morphotypes of Bs within the areal. Almost all individuals have Bs. Their number and morphology can be different, and reach up to 30. Now, the accumulated material allows us to draw conclusions not only regarding the general patterns of Bs distribution (Vujošević *et al*., 2018; Borisov, Zhigarev, 2018), but also to identify changes in these patterns over time (Borisov, 2008; Zhigarev *et. al*., 2022).

**Materials and Methods**

The karyotype was determined by standard methods (Borisov, Zhigarev, 2018) of 153 *A. peninsulae* individuals caught in the northern part of Lake Teletskoye (Mountain Altai) over a 36-year period (1978–2014) and 84 individuals from the Southern Baikal region over a 35-year period (1986–2019). Trapping was carried out unevenly, with a step of 3-12 years.

**Results**

In the Southern Baikal region, low variability of statistical indicators of Bs systems was shown over 35 years of observations, as well as the absence of significant differences between samples of different years. The average number of Bs is from 8.11±0.9 to 9.14±0.4, with the relative constancy of their morphological characteristics (Zhigarev *et. al*., 2022).

In Mountain Altai, on the contrary, a pronounced directional dynamic was observed over 36 years. Three periods can be distinguished. From 1978 to 2002 there was a rapid growth in the number of Bs, with a relatively uniform average increase of 1.4 chromosomes per decade from 3.17±0.2 to 6.5±0.5. In 2002–2012, a period of stabilization began, at a more than two-fold high level compared to the beginning of the 80s (the differences are significant (t>6.7, compared with all years of the stabilization period). Throughout the decade, the average number of chromosomes has changed in a narrow range - from 6.31 to 6.88, which probably indicates the achievement of the population maximum of the Bs level of this population under real environmental conditions. Then (2012–2014), there was a tendency for a decrease in the number of Bs, to 4.9±0.36 (t=2.14; p=0.04) The stages of growth, stabilization and decline correspond to the dynamics of morphology, their conditional "mass" and other indicators characterizing the Bs system.

**Conclusions**

Thus, long-term observations indicate the presence of population-specific changes in Bs systems in the species *A. peninsulae*, as well as the still unclear and hidden patterns of their manifestation in specific populations.

**References**

1. Vujošević M, Rajičić M, Blagojević J. B Chromosomes in Populations of Mammals Revisited. Genes 2018; 9(10):487.
2. Borisov YM, Zhigarev IA. B Chromosome system in the Korean field mouse *Apodemus peninsulae* Thomas 1907 (Rodentia, Muridae) Genes 2018; 9:472.
3. Borisov YM. Increase in the number of the B-chromosomes and variants of their system in mouse *Apodemus peninsulae* in Mountain Altai population over 26 years. Russ. J. Genet. 2008; 44:1070–1079.
4. Zhigarev IA, Kryshchuk IA, Borisova ZZ, Borisov YuM. Stability of the population system of B chromosomes of the Korean field mouse *Apodemus peninsulae* (Mammalia, Rodentia) in the Southern Baikal Region: 35 Years of Observations. Russ. J. Genet. 2022; 58(5):558–565.

## Session 3 - B chromosome effects

### S3-PL1 B chromosomes in the RNA world

#### Jordana IN Oliveira^1,2^, Diogo C Cabral-de-Mello^3^, Guilherme T Valente^1^, Cesar Martins^1^

##### ^1^Department of Structural and Functional Biology, Institute of Bioscience at Botucatu, Sao Paulo State University (UNESP), Botucatu, SP, Brazil; ^2^Department of Biology, Faculty of Science, University of Ottawa, ON, Canada; ^3^Department of General and Applied Biology, Institute of Biosciences, Sao Paulo State University (UNESP), Rio Claro, SP, Brazil

###### **Correspondence:** Cesar Martins (cesar.martins@unesp.br)

*BMC Proceedings* 2023, **17(20)**:S3-O1

B chromosomes are supernumerary elements found in diverse eukaryotes, including fungi, plants, and animals. They originate during errors in meiosis and are composed of duplicated sequences from the host genome. Although they are mostly heterochromatic and low dense in coding genes, the B chromosome cells transcribe sequences and can impact the expression of host genomes. In this review, we draw a timeline of studies investigating B chromosomes and RNAs, showing the advances and main findings during the recent history of B chromosome science. Also, we highlighted which RNA classes have already been reported in the B chromosomes and which ones could be more explored in the focus of new perspectives to understand the complex B chromosome biology. In this way, we presented a B chromosome tree (B-tree) of life, indicating which organism branches already have had their functional RNA studied for the B chromosome. We suggest the investigation of other unexplored RNA classes and types of functional analysis combined with cytogenetics studies to complete the B-tree of life from an RNA perspective.

**Acknowledgements**

This work was funded by grant number 2015/16661-1 - Fundação de Amparo à Pesquisa do Estado de São Paulo (FAPESP).

### S3-PL2

*BMC Proceedings* 2023, **17(20)**:S3-O2

https://rdcu.be/dnH0u

### S3-PL3 Frequent horizontal transfer of an accessory chromosome increases the fitness of an asexual entomopathogenic fungus

#### Michael Habig^1,2^, Anna Grasse^3^, Eva H Stukenbrock^1,2^, Hanna Leitner^3^, Sylvia Cremer^3^

##### ^1^University of Kiel, Germany; ^2^Max Planck Institute for Evolutionary Biology, Germany; ^3^ISTA, Austria

###### **Correspondence:** Michael Habig (mhabig@bot.uni-kiel.de)

*BMC Proceedings* 2023, **17(20)**:S3-O3

Accessory chromosomes in fungi display presence/absence polymorphism and are non-essential, yet they can confer a fitness advantage. Horizontal transfer of accessory chromosomes has long been considered unlikely because full-length chromosomes are generally thought to be inherited vertically from one generation to the next. However, recent genomic comparisons have challenged this assumption, suggesting that such transfers may have enhanced fitness and host specificity in the past. Nevertheless, the prevalence and extent of horizontal transfer of entire chromosomes remain poorly understood. Here, we report frequent horizontal transfers of accessory chromosomes in the asexual entomopathogenic fungus *Metarhizium robertsii* during the co-infection of insect hosts. Surprisingly, we find that only the accessory chromosome is transferred between distinct strains, with no transfer of other donor chromosomes. The recipient strain that acquires the accessory chromosome gains a competitive advantage over the ancestral strain, indicating improved fitness. Moreover, we demonstrate that the same accessory chromosome was horizontally transferred in the field between *M. robertsii* and another insect pathogen, *M. guizhouense*, representing the first reported case of an inter-species horizontal chromosome transfer. The transferred accessory chromosome contains genes encoding two putative histones and multiple histone-modifying enzymes, which may contribute to its preferential transfer across species boundaries. In summary, our findings provide evidence that a specific accessory chromosome is frequently transferred within the *Metarhizium* genus in both controlled experimental conditions and also in the field. Such horizontal transfers of full-length chromosomes may represent a comparatively common mechanism for gene exchange and the acquisition of presumably fitness-relevant genes in otherwise asexual fungal pathogens.

### S3-O1 Sex differentiation genes expression is influenced by B chromosomes in the genus *Psalidodon* (Teleostei, Characiformes)

#### Mateus Rossetto Vidal^1^, Duilio Mazzonni Zerbinato de Andrade Silva^2^, Lucas Fortino Lasmar^1^, Ricardo Utsunomia^3^, Thalles Fernando Rocha Ruiz^4^, Sebastião Roberto Taboga^4^, Claudio Oliveira^1^, Fausto Foresti^1^

##### ^1^Department of Structural and Functional Biology, Biosciences Institute, São Paulo State University,Botucatu, São Paulo, Brazil; ^2^Department of Biological Sciences, School of Sciences, São Paulo State University, Bauru, São Paulo, Brazil; ^3^School of Pharmaceutical Sciences, University of Campinas, Campinas, São Paulo, Brazil; ^4^Department of Biology, Institute of Biosciences, Humanities and Exact Sciences, São Paulo State University, São José do Rio Preto, São Paulo, Brazil

###### **Correspondence:** Mateus Rossetto Vidal (mateus.vidal@unesp.br)

*BMC Proceedings* 2023, **17(20)**:S3-O4

The tetra fish group *Psalidodon* has macro-B chromosomes which share the same origin at least in four species. Among them, the B chromosome of *P. scabripinnis* is related to changes in reproductive cycle. Also, *P. paranae* presents a high-frequency B chromosome preferentially found in females. However, no apparent effects in sex-related mechanisms are seen in *P. fasciatus* and *P. bockmanni*. Thus, we aimed to analyse whether B chromosomes influence expression of genes involved in sex differentiation in *Psalidodon* species. For this, female adults of *P. fasciatus*, *P. bockmanni* and *P. paranae*, and *P. paranae* males were collected in São Paulo state rivers, Brazil. 0B and 1B individuals were identified by classical cytogenetics analysis. RT-qPCR analysis was performed in gonad tissues for the main genes involved in sex differentiation, i.e., *amh, cyp19a1a, dmrt1, esr1, sox9,* and *nobox,* using the 2^−ΔΔCt^ method with *tbp*, *ppiaa*, *pgk1* and *hprt1* as reference genes. We performed immunohistochemical analysis with CYP-19 and ER-alpha antibodies to validate RT-qPCR results. Statistical analyses were performed using two-group comparisons by Gardner-Altman estimation plot method. In *P. paranae* 1B females, two genes involved in the estrogen pathway (*cyp19a1a* and *esr1*), as well as their respective proteins (CYP19 and ER-alpha), were subexpressed, while the *nobox* gene, essential to oogenesis process, was overexpressed. In *P. paranae* 1B males, *dmrt1* and *nobox* genes were overexpressed. In contrast, in 1B animals of the other two species, our results showed nearly significant (P=0.069) *cyp19a1a* overexpression in *P. bockmanni* and nearly significant subexpression of *esr1* in *P. fasciatus* (P=0.058). We hypothesize that subexpression of estrogen pathway in *P. paranae* 1B females could lead to a delayed reproductive peak, such as observed for *P. scabripinnis*, due to delayed gonadal development. Furthermore, overexpression of *dmrt1* in *P. paranae* 1B males might be associated with extension in the reproductive peak, as observed for *P. scabripinnis*. Moreover, a higher frequency of 1B females associated with *nobox* gene overexpression in *P. paranae* could lead to greater efficiency in oogenesis, thus increasing reproductive success. In contrast, *P. fasciatus* and *P. bockmanni*, which present low B chromosome frequency, only a moderate influence was observed. This might indicate that influences in sex-related gene expression could be related to increased frequencies of *Psalidodon* B chromosomes. In conclusion, our results suggest that mechanisms for manipulation of sex-related gene expression might have originated in an ancestral B chromosome and then were neutralized in *P. fasciatus* and *P. bockmanni*.

**Acknowledgements**

This research was funded by FAPESP, CAPES and CNPQ.

### S3-O2 Unravelling the role of the germline-restricted chromosome in the course of zebra finch development using Spatial Transcriptomics and RNA-seq

#### Niki Vontzou^1,2^, Francisco J Ruiz-Ruano Campana^1,2^, Simone Immler^1^, Alexander Suh^1,2,3^

##### ^1^School of Biological Sciences, University of East Anglia, Norwich Research Park, Norwich, Norfolk,UK; ^2^Centre for Molecular Biodiversity Research, Leibniz Institute for the Analysis of Biodiversity Change, Museum Koenig Bonn,Bonn, Germany; ^3^Department of Organismal Biology – Systematic Biology, Evolutionary Biology Centre, Science for Life Laboratory, Uppsala University, Uppsala, Sweden

###### **Correspondence:** Niki Vontzou (n.vontzou@uea.ac.uk), Alexander Suh (a.suh@leibniz-lib.de)

*BMC Proceedings* 2023, **17(20)**:S3-O5

More than two thirds of all bird species have a mysterious chromosome which is solely present in their germline, as it is excluded from the soma with an unknown mechanism and at an unknown time point during early embryogenesis. In the zebra finch *Taeniopygia guttata*, this germline-restricted chromosome (GRC) constitutes the largest chromosome of the genome. The zebra finch GRC was initially cytogenetically detected back in the late 1990s, yet its genetic content has only been thoroughly examined in the last five years. The genetic content of the zebra finch GRC suggests some selfish B-chromosome-like attributes, since it consists of paralogous sequences duplicated from other chromosomes, with some of these paralogs further duplicated into hundreds of copies on the GRC. Whether the GRC evolved from a parasitic B chromosome and subsequently acquired essential genes for the germline is yet to be elucidated. Although gene expression analyses of the zebra finch GRC suggest a germline-specific function of some genes moved onto the GRC, currently available transcriptomic data are solely restricted to adult gonads. Hence, the gene expression profiles of the GRC paralogs for different gonad developmental and embryonic stages still remain unknown. In this project, we use RNA-seq and Spatial Transcriptomics to produce a high-resolution GRC expression map across the zebra development, exploring the GRC-linked expression on and after oviposition as well as on and after hatching day. We demonstrate the upregulation of *tfeb* GRC paralog on oviposition, a regulator of the pluripotency transcriptional network, suggesting a potential role of the GRC in sustaining the pluripotency in the zebra finch embryonic germline. Moreover, we highlight the overall upregulation of GRC paralogs in the hatchling gonad in both females and males when compared to the adult gonad. Our results unravel the germline-specific functions of the GRC and support it may serve as a ‘domesticated’ B chromosome in the zebra finch genome.

**Acknowledgements**

We thank the European Research Council (grant 101002158 GermlineChrom to A.S.), the Marie Skłodowska-Curie Individual Fellowships (grant 875732 to F.J.R.-R.), the Swedish Research Council Vetenskapsrådet (grant 2020–04436 to A.S.), the Max Planck Institute for Biological Intelligence (access to birds and laboratories by Yifan Pei, Wolfgang Forstmeier, Bart Kempenaers), the National Genomics Infrastructure in Stockholm (sample preparation and sequencing by Renuka Kudva, Xesús Abalo, Annelie Mollbrink, Jörg Bachmann) funded by the Science for Life Laboratory, and University of East Anglia (PhD fellowship to N.V.; practical training by Andrea Münsterberg, Emily Smith).

## Session 4 - Segregation behaviour of B chromosomes

### S4-PL1 Drive and Genomic Conflict of the Maize B Chromosome

#### Hua Yang^1^, Hua Liu^2^, Patrice Albert^1^, Wayne Carlson^2^, Jian Liu^3^, Zach Loschinskey^3^, Jianlin Cheng^3^, Bing Yang^2^, James A. Birchler^1^

##### ^1^Division of Biological Sciences, University of Missouri, Columbia, USA; ^2^Division of Plant Science and Technology, University of Missouri, Columbia, USA; ^3^Department of Electrical Engineering and Computer Science, University of Missouri, Columbia, USA

###### **Correspondence:** James A. Birchler (BirchlerJ@Missouri.edu)

*BMC Proceedings* 2023, **17(20)**:S4-O1

The B chromosome of maize is a nonvital chromosome that persists in populations by a drive mechanism. This consists of nondisjunction at the second pollen mitosis that produces the two sperm followed by the sperm with the B chromosomes showing preferential fertilization of the egg compared to the central cell in the process of double fertilization. The nondisjunction occurs at the centromere while other sites on the B chromosome are required in trans for it to occur. The sequence of the B chromosome was recently completed by an international group. Chromosomal rearrangement breakpoints involving the B chromosome were placed on the reference sequence. Using translocations of the B chromosome with the short arm of chromosome 9 (TB-9Sb), which had been subjected to EMS treatment and which have lost the nondisjunction property, sequence comparisons implicated B gene 666 as a trans acting factor. Three CRISPR-Cas9 edits of this gene eliminated nondisjunction on a phenotypically marked normal B chromosome. Computational modelling of the predicted protein of gene 666 suggests it is a receptor for ubiquitination targets specifically for replication protein A subunits, which are involved in DNA replication and repair. DNA read counts along the B chromosome from mature pollen in normal TB-9Sb and those mutant for nondisjunction indicate a difference in replication of the B specific repeats in and around the B centromere and the centromere adjacent knob heterochromatic repeats. Together the results suggest that nondisjunction involves a modification of the replication dynamics of highly repetitive sequences at the centromeric region of the B chromosome. In the High Loss background of maize, B chromosomes also cause the heterochromatic knobs on the normal A chromosome to remain adhered at the second pollen mitosis, which causes chromosomal breakage of those chromosomes and de-silences transposable elements. In this background, the B chromosomes can also cause all chromosomes to remain adhered resulting in diploid sperm that are functional. Thus, while the drive mechanism of the B chromosome allows it to be perpetuated in populations, it causes a conflict with the normal genome in some backgrounds.

**Acknowledgement**

Research was supported by a grant from the National Science Foundation (USA).

### S4-O1 Identification of the *trans*-acting element that controls the non-disjunction of the rye B chromosome

#### Jianyong Chen^1^, Anastassia Boudichevskaia^1,2^, Mark Timothy Rabanus-Wallace^1^, Anna Voigt^1^, Zuzana Tulpova^3^, Helena Toegelová^3^, Hana Simkova^3^, Thomas Schmutzer^4^, Jiri Macas^5^, Katharina Bürstenbinder^6^, Jan Bartos^3^, Andreas Houben^1^

##### ^1^Leibniz Institute of Plant Genetics and Crop Plant Research (IPK), Gatersleben, Seeland, Germany; ^2^KWS SAAT SE & Co. KGaA, Einbeck, Germany; ^3^Institute of Experimental Botany of the Czech Academy of Sciences, Olomouc, Czech Republic; ^4^Martin Luther University Halle-Wittenberg, Institute for Agricultural and Nutritional Sciences, Halle (Saale), Germany; ^5^Biology Centre, Czech Academy of Sciences, Ceske Budejovice, Czech Republic; ^6^Department of Molecular Signal Processing, Leibniz Institute of Plant Biochemistry, Halle (Saale), Germany

###### **Correspondence:** Jianyong Chen (chenj@ipk-gatersleben.de), Andreas Houben (houben@ipk-gatersleben.de)

*BMC Proceedings* 2023, **17(20)**:S4-O2

**Background**

The B chromosome is a dispensable element in the genome of many plants, animals, and fungi. To counteract the elimination of supernumerary chromosomes, many B chromosomes evolved a drive mechanism to transmit themselves at a higher frequency compared to the standard A chromosome. To decipher the chromosome drive process, we have selected the B chromosome of rye (*Secale cereale*) as a model. During the first pollen grain mitosis (PMI), rye B sister chromatids continue to stay cohesive rather than separate, and then both chromatids preferentially enter the generative nucleus. The drive process of the rye B is controlled by the *trans*-acting B-located non-disjunction control region. Bs lacking the non-disjunction control region (NCR) undergo normal disjunction at the first pollen mitosis and therefore do not drive. The rye B shows an efficient drive also when it is introduced into wheat.

**Materials, Methods, and Results**

To narrow down the non-disjunction control region, we compared the repeat composition of the long arm of different B variants by fluorescence *in situ* hybridization (FISH) and identified a subtelomeric region (~10% of the rye B chromosome) that controls the drive of the rye B chromosome. To identify the genetic element(s) that control the non-disjunction of the rye B chromosome, we assembled the rye B sequences using PacBio, Hi-C, Nanopore sequencing, and optical mapping. In addition, 33 RNA-seq data sets from anthers undergoing PMI of rye and wheat were generated, including of drive-positive and drive-negative specimens. Differential expression analysis identified that only 16 candidates are up-regulated in all the comparisons between drive-positive and drive-negative data. PCR-based mapping revealed that 10 of the 16 candidates were located within the NCR. Their tissue- and stage-specific expression patterns were tested in RNA-seq data from 7 different tissues of wheat with 2B chromosomes. The drive-associated candidate gene *NCR28* shows a PMI-specific and strong expression. *NCR28* is encoded by 13 transcriptionally active copies, and FISH confirmed the NCR-specific localization and its multiple copy number. Transient expression of an EYFP-NCR28 construct in *Nicotiana benthamiana* revealed a microtubule-like pattern in dividing cells. Our results shed light on the mechanism of chromosome drive in plants.

**Conclusions and outlooks**

A gene cluster *NCR28* which is specifically expressed during PMI was identified as the candidate that controls the drive of the rye B chromosome. *NCR28* is a microtubule-associated protein that participates in cell division. Next, CRISPR-Cas9 will be applied to study further the function of NCR28 in the process of chromosome drive.

**Acknowledgments**

We thank the China Scholarship Council (CSC202006850005), Deutsche Forschungsgemeinschaft (DFG) project HO1779/30-1, European Regional Development Fund (No. CZ.02.1.01/0.0/0.0/16_019/0000827) and IPK Gatersleben for funding and T. Endo (Japan) for providing the valuable seeds.

### S4-O2 Wild sorghum B chromosome: a forgotten source of biological abnormalities

#### Karafiátová Miroslava^1^, Bojdová Tereza^1^, Malurová Magdaléna^1^, Kumar Vinod^2^, Bartoš Jan^1^

##### ^1^Institute of Experimental Botany of the Czech Academy of Sciences, Centre of Plant Structural and Functional Genomics, Olomouc, Czech Republic; ^2^Institute of Experimental Botany of the Czech Academy of Sciences, Laboratory of Pollen Biology, Prague 6 – Lysolaje, Czech Republic

###### **Correspondence:** Karafiátová Miroslava (karafiatova@ueb.cas.cz)

*BMC Proceedings* 2023, **17(20)**:S4-O3

B chromosomes are generally considered as mysterious genome components and the one in wild sorghum *S. purpureosericeum* is unequivocal proof. The behaviour of this B chromosome involves abnormalities across whole plant growth from the developing embryo to mature pollen grain. The embryonic tissue differentiation is accompanied by B chromosome elimination from most of the embryonal organs/tissues in early development. This process is mirrored in a very specific distribution in the adult plant, where B chromosomes are preserved consistently only in reproductive organs including all somatic tissues of the panicle. Besides embryonal tissues, we observed the active elimination of the B chromosome from all somatic tissues, where B chromosome was detected and thus we assume that some level of B chromosome depletion may be a part of the developmental programme of all those tissues. Aside from B chromosome elimination, two mechanisms of its maintenance were discovered. In wild sorghum, Bs can be accumulated either via the segregation failure at first pollen division, or via the extraordinary pathway of polymitosis in the pollen grain, where the B chromosome non-disjoins during the extra division of the vegetative nucleus. This alternative pathway produces multicellular pollen grains carrying an extra nucleus, which are viable and capable of germination.

**Acknowledgements**

The work was supported by The Czech Science Foundation (grant no. 22-02108S) and by The Czech Academy of Sciences (project no. DAAD-22-02).

### S4-O3 GRC elimination during embryogenesis in zebra finch

#### Lyubov Malinovskaya^1^, Tatiana Bikchurina^2^, Pavel Borodin^1^, Anna Torgasheva^1^

##### ^1^Institute of Cytology and Genetics SB RAS, Novosibirsk, Russia; ^2^Novosibirsk State University, Novosibirsk, Russia

###### **Correspondence:** Lyubov Malinovskaya (l.malinovskaia@g.nsu.ru)

*BMC Proceedings* 2023, **17(20)**:S4-O4

**Background**

Recently discovered in songbirds, the germline-restricted chromosome (GRC) is sometimes considered a peculiar example of B chromosomes (Bs). Both GRC and Bs are supernumerary in addition to the basic set of A chromosomes. However, compared to Bs, which show drastic differences in copy number between different organs and tissues in the same species, the behaviour of GRC appears to be more programmed. GRC is present in germline cells and absent in somatic cells. During spermatogenesis, GRC is eliminated from primary spermatocytes and forms a micronucleus that is later destroyed in the cytoplasm. The absence of GRC in somatic cells suggests that GRC is eliminated early in embryogenesis. However, the time and pace of this process remain unclear.

**Materials and Methods**

To investigate the GRC behaviour during early embryogenesis, we prepared cryosections of gelatin-embedded zebra finch (*Taeniopygia guttata*) embryos at different stages. For GRC detection, we performed FISH with a GRC-specific microdissection probe.

**Results**

In embryos from freshly laid eggs (stage EGK.VI), we detected a few GRC-positive cells that were dispersedly located. This indicates that elimination of GRC from somatic cells is initiated during the first 24 hours after fertilization. In cytoplasm of some cells at this stage, we visualized GRC-positive micronuclei similar to those in spermatocytes. This observation suggests that the mechanism of GRC elimination from somatic cells might be similar to those observed in spermatogenesis. No GRC-positive micronuclei were detected after formation of the primitive streak at stage HH2 (22-24 hours of incubation). This indicated that GRC elimination might have been completed by this time.

**Conclusions**

Our findings demonstrate that zebra finch GRC is rapidly eliminated from somatic cells and is restricted by the germ line, presumably, at stage HH2. The mechanism of GRC elimination from somatic cells is, apparently, similar to the mechanism of GRC elimination from spermatocytes.

**Acknowledgements**

The study was supported by the Russian Science Foundation (project No. 23-14-00182).

### S4-O4

*BMC Proceedings* 2023, **17(20)**:S4-O5

https://www.pnas.org/doi/full/10.1073/pnas.2103960119

### S4-P1 Epigenetic landscape of B chromosome in *Pseudoccocus viburni*

#### Marion Herbette, Laura Ross

##### University of Edinburgh, Institute of Ecology and Evolution, UK

###### **Correspondence:** Marion Herbette (mherbett@ad.ac.uk)

*BMC Proceedings* 2023, **17(20)**:S4-P1

B chromosomes are maintained in populations through a transmission advantage provided by diverse cellular mechanisms. Here we study a B chromosome in mealybug, which has an unusual reproductive system, known as paternal genome elimination, in which males recognise, silence, and discard the set of chromosomes inherited from their fathers during male meiosis. Only the maternally inherited chromosomes are passed on to the offspring. In the mealybug species *Pseudococcus viburni*, "selfish" B chromosomes are present and have evolved a way to escape paternal genome elimination. This allows them to be transmitted to the offspring regardless of their parental origin. This study aims to better understand the mechanism underlying the ability of B chromosomes to escape genome exclusion, as well as the molecular mechanisms responsible for paternal genome elimination. Based on previous cytological studies, we hypothesized that the B chromosomes escape exclusion by altering their chromatin structure to mimic that of the maternal genome. We aim to determine which epigenetic marks are involved in this structural change. To achieve this, we first analysed gene expression between lines with and without the B chromosome. These analyses allowed us to identify interesting candidate genes associated with chromatin regulation. We found a histone acetyl transferase responsible for acetylation of H3K56, which is encoded on the B chromosome and overexpressed in males during spermatogenesis. We also examined genes encoding histone-modifying enzymes expressed during spermatogenesis, which could be involved in imprinting and the escape mechanism. Subsequently, we conducted a cytogenetic study of histone marks during male meiosis and have identified histone marks of interest that differentially decorate the maternal or paternal genome and are also present on B chromosomes. Additionally, we analysed the behaviour of the meiotic spindle and the attachment of the B chromosome during meiosis I and II.

### S4-P2 Identification and functional characterization of genes controlling the process of root-specific B chromosome elimination in *Aegilops speltoides*

#### Gihwan Kim, Johannes Thiel, Jedrzej Jakub Szymanski, Jörg Fuchs, Mohammad-Reza Hajirezaei, Suriya Amutha, Stefan Heckmann, Andreas Houben

##### Leibniz Institute of Plant Genetics and Crop Plant Research (IPK), Seeland, Germany

###### **Correspondence:** Gihwan Kim (kimg@ipk-gatersleben.de)

*BMC Proceedings* 2023, **17(20)**:S4-P2

Genome stability is a crucial feature of eukaryotic organisms since the loss or gain of even a single chromosome can significantly affect normal development and the organism as a whole. Nonetheless, some organisms tend to eliminate specialized chromosomes (such as sex or B chromosomes) either partially or entirely in a certain stage of development. B chromosomes are present in some eukaryotic organisms exclusively in addition to standard A chromosome complement. Whereas some organisms carry B chromosomes in every tissue, others do not have B chromosomes in some organs or tissues. The presence or absence of the B chromosome is variable but also depends on the developmental stage of the organism, e.g., the early stage of embryogenesis. The elimination of B chromosomes in goatgrass *Aegilops speltoides*, is root-specific and the elimination undergoes at the onset of embryogenesis (embryo six days after pollination). Aided by technical advances in microscopic analysis, genome sequencing, and bioinformatics analysis, the mechanism behind B chromosome elimination in *Aegilops speltoides* is beginning to unravel; however, the genes controlling this mechanism remain highly elusive.

In this research, we aim to identify the candidate genes that control root-specific B chromosome elimination. Genome stability is a crucial feature of eukaryotic organisms since the loss or gain of even a single chromosome can significantly affect normal development and the organism as a whole. Nonetheless, some organisms tend to eliminate specialized chromosomes (such as sex or B chromosomes) either partially or entirely in a certain stage of development. B chromosomes are present in some eukaryotic organisms exclusively in addition to standard A chromosome complement. Whereas some organisms carry B chromosomes in every tissue, others do not have B chromosomes in some organs or tissues. The presence or absence of the B chromosome is variable but also depends on the developmental stage of the organism, e.g., the early stage of embryogenesis. The elimination of B chromosomes in goatgrass *Aegilops speltoides*, is root-specific and the elimination undergoes at the onset of embryogenesis (embryo 6 days after pollination). Aided by technical advances in microscopic analysis, genome sequencing, and bioinformatics analysis, the mechanism behind B chromosome elimination in *Aegilops speltoides* is beginning to unravel; however, the genes controlling this mechanism remain highly elusive.

In this research, we aim to identify the candidate genes that control root-specific B chromosome elimination process by comparative RNA-seq analysis of the transcriptome from tissues undergoing B chromosome elimination compared to the same tissue from plants that do not carry B chromosomes. Primarily, pooled B-positive and B-negative materials which are young embryos (6-8 days after pollination), the laser-captured microdissected meristematic region from the mid-aged embryos (17-20 days after pollination), and adventitious root buds were compared to identify candidate transcripts. Moreover, compared transcripts from the tissues without the B chromosome elimination phenomenon, such as young leaves, roots, and mature embryos were subtracted from the candidate transcripts. Co-expression analysis of candidate genes will be performed to identify new putative regulators of B chromosome elimination. Finally, the function of candidate genes will be characterized using virus-induced gene silencing.

By comparative RNA-seq analysis of the transcriptome from tissues undergoing B chromosome elimination compared to the same tissue from plants that do not carry B chromosomes. Primarily, pooled B-positive and B-negative materials which are young embryos (6-8 days after pollination), the laser-captured microdissected meristematic region from the mid-aged embryos (17-20 days after pollination), and adventitious root buds were compared to identify candidate transcripts. And compared transcripts from the tissues without the B chromosome elimination phenomenon such as young leaves, roots, and mature embryos were subtracted from the candidate transcripts. Co-expression analysis of candidate genes will be performed to identify new putative regulators of B chromosome elimination. Finally, the function of candidate genes will be characterized using virus-induced gene silencing.

**Acknowledgements**

This work was supported by the Deutsche Forschungsgemeinschaft DFG - Project number 498976470.

### S4-P3 Characterization of candidate genes controlling the drive of a B chromosome

#### Taoran Liu, Jianyong Chen, Andreas Houben

##### Leibniz Institute of Plant Genetics and Crop Plant Research (IPK), Seeland, Germany

###### **Correspondence:** Taoran Liu (liut@ipk-gatersleben.de)

*BMC Proceedings* 2023, **17(20)**:S4-P3

**Background**

B chromosomes (Bs) are not required for the regular growth and development of organisms, yet they have been discovered in all eukaryotic phyla. Most Bs persist in populations by using the cellular machinery required from the inheritance and maintenance of A chromosomes but offer no advantages to the organisms. Therefore they are considered parasitic and selfish elements. To maintain chromosome transmission, many Bs evolved a drive mechanism that does not conform to Gregor Mendel's genetic law. In some species, they can even exceed the number of standard A chromosomes (As). Even though the drive is one of the most important features of many Bs, insights about the drive mechanism exist at the cellular level only for a few species. Aided by technical advances in PacBio, Hi-C, Nanopore sequencing, optical mapping, and RNA-seq, the mechanism behind the drive of Bs in rye is beginning to unravel. In rye, the B chromosome drive-associated candidate gene *NCR28* shows a strong pollen grain mitosis-specific expression. However, the functional mechanism of how *NCR28* regulates chromosome drive is unknown. In this research, we aim to analyse the function of *NCR28* by gene activity modulation, microtubule-binding experiments, and other different molecular experiments, using rye, barley, and *Arabidopsis thaliana* as models.

An *in vitro* test will be applied to determine tubulin-binding differences between the B- and A-encoded *NCR28* variants to test the hypothesis that overexpression of the B-encoded *NCR28* (co)modulates the segregation dynamics (drive) of the B chromosome directly or indirectly. Since no experimental data exists for the function of *NCR28*, we aim to analyse *NCR28* homologous genes function in barely and *A. thaliana*, by using virus-induced gene silencing (VIGS), overexpression, and other different molecular experiments.

**Acknowledgements**

We thank the Deutsche Forschungsgemeinschaft (DFG) (HO1779/30-2) for funding.

## Session 5 - Origin and evolution of B chromosomes

### S4-PL1 To B, or not to B, that is the question of the germline-restricted chromosome of passerine birds

#### Alexander Suh^1,2,3^

##### ^1^Centre for Molecular Biodiversity Research, Leibniz Institute for the Analysis of Biodiversity Change, Museum Koenig Bonn, Bonn, Germany; ^2^Institute of Evolutionary Biology and Ecology, University of Bonn, Bonn, Germany; ^3^Department of Organismal Biology – Systematic Biology, Evolutionary Biology Centre, Science for Life Laboratory, Uppsala University, Uppsala, Sweden

###### **Correspondence:** Alexander Suh (a.suh@leibniz-lib.de)

*BMC Proceedings* 2023, **17(20)**:S4-O1

The over 6000 species of passerine birds all likely exhibit a germline-restricted chromosome (GRC) which is present in one copy in male germline cells, in two copies in female germline cells, and absent from somatic cells. Recent advances in cytogenetics and genomics have finally made studying this peculiar form of programmed DNA elimination possible, as highlighted by the several contributed talks and posters about the passerine GRC at this conference. Here I summarize the history of GRC research and the manifold questions and confusions that have arisen so far. Please decide for yourself: Is the GRC (still) a B chromosome?

**Acknowledgements**

I would like to thank all past and present GRC researchers, all collaborators, and all participants of the GRC brainstorming meetings. My research group is currently supported by the European Research Council (101002158 GermlineChrom) and the Swedish Research Council Vetenskapsrådet (2020–04436).

### S4-PL2 B chromosomes and extrachromosomal DNA

#### Vladimir A Trifonov^1^, Dunja K Lamatsch^1^, Alex I Makunin^2^

##### ^1^Research Department of Limnology, University of Innsbruck, Austria; ^2^Wellcome Sanger Institute, Hinxton, United Kingdom

###### **Correspondence:** Vladimir A Trifonov (Vladimir.Trifonov@uibk.ac.at)

*BMC Proceedings* 2023, **17(20)**:S4-O2

B chromosomes and extrachromosomal DNA (ecDNA) represent two manifestations of eukaryotic genome redundancy, and they share many common traits: both represent a non-essential genomic fraction that occurs in many eukaryotic species and is mostly derived from genomic fragments of the standard genome. While B chromosomes usually contain functioning centromeres and standard telomeres, maintaining their stability in cell divisions, ecDNA lacks those. Recently, with the development of sequencing techniques and analysis, more data became available on ecDNA, and it was found in the representatives of all major eukaryotic groups and in different tissues and developmental stages. Previously, we have found that some genomic fragments occur particularly often in B chromosomes. We assume that the same genomic regions may be overrepresented in the extrachromosomal DNA. These regions may be particularly unstable and prone to amplification, and as for transcribed genes - the dosage sensitivity must also play a role. We propose that ecDNA may provide a substrate for B chromosome origin and participate in the evolution of already existing B chromosomes by providing additional genomic segments and ectopic regulatory sequences. The study of ecDNA turnover will be important not only for understanding B chromosome origin but also for estimating the causes of genomic instability in cancer.

### S4-O1 Germline-restricted chromosome in songbirds

#### Anna Torgasheva, Lyubov Malinovskaya, Kira Zadesenets, Nikolai Rubtsov, Pavel Borodin

##### Institute of Cytology and Genetics SB RAS, Prosp. Lavrentieva, 10, 630090, Novosibirsk, Russia

###### **Correspondence:** Anna Torgasheva (torgasheva@bionet.nsc.ru)

*BMC Proceedings* 2023, **17(20)**:S4-O3

**Background**

Germline-restricted chromosome (GRC) was discovered in zebra finch by Maria Ines Pigozzi and Alberto Solari 25 years ago and for a long time was considered as the only case of B chromosomes in birds. We recently showed that GRC is apparently present in at least half of bird species and it is probably originated in a common ancestor of songbirds. GRC is absent in somatic cells and eliminated from spermatids. Unlike B chromosomes, GRC is indispensable element of germline since no meiotic cell without GRC has been detected to date. Despite major interest in the topic, the GRC origin, function, evolution, and mechanisms of inheritance and elimination remain unknown.

**Materials and Methods**

To investigate the diversity and identify evolutionary features of GRC we analysed its meiotic behaviour in more than 20 songbird species using immunolocalization of key meiotic proteins. We performed interspecies FISH with microdissected GRC-specific probes to assess the degree of homology between different species.

**Results**

We demonstrated that GRC shows remarkable variation in size and genetic content. The size of GRC varies from a dotted microchromosome to the largest macrochromosome in the karyotype (up to 10% of genome) and do not show phylogenetic clustering. Inter-species FISH showed no or little homology between different taxa of songbirds and demonstrated that GRC tends to accumulate large number of repeated DNA sequences, different in different species. Variation in the number of copies, typical of B chromosomes, is also characteristic of GRC, although to a lesser extent. While males in most species usually contain one GRC copy and females - two, in several species, we found polymorphism and mosaicism for the GRC copy number in females and males. These results allowed us to suggest hypotheses about the possible mechanisms of GRC inheritance.

**Conclusions**

In contrast to highly conservative and compact bird genomes, GRC is extremely variable and tends to accumulate a large number of repeated DNA sequences. Its evolution is accompanied by rapid turnover of sequences homologous to different regions of A-chromosomes. GRC exhibits some features similar to those of B chromosomes. However, it apparently acquired sequences critical for germline development and spermatogenesis and/or ensuring its own transmission via mitotic and meiotic divisions.

**Acknowledgements**

The research was funded by the Russian Science Foundation (grant #23-14-00182).

### S4-O2 The germline-restricted chromosome in passerine birds: a possible example of B chromosome “cellular domestication”

#### Radka Reifová^1^, Manuelita Sotelo-Muñoz^1^, Stephen A. Schlebusch^1^, Ondřej Kauzál^2,3^, Dmitrij Dedukh^4^, Manon Poignet^1^, Jakub Rídl^1^, Karel Janko^4^, Alexander Suh^5,6^, Tomáš Albrecht^1,2^

##### ^1^Department of Zoology, Faculty of Science, Charles University, Prague, Czech Republic; ^2^Institute of Vertebrate Biology, Czech Academy of Sciences, Czech Republic; ^3^Department of Ecology, Faculty of Science, Charles University, Prague, Czech Republic; ^4^Institute of Animal Physiology and Genetics, Czech Academy of Sciences, Czech Republic; ^5^School of Biological Sciences, University of East Anglia, Norwich, UK; ^6^Department of Organismal Biology, Uppsala University, Uppsala, Sweden

###### **Correspondence:** Radka Reifová (radka.reifova@natur.cuni.cz)

*BMC Proceedings* 2023, **17(20)**:S4-O4

The germline-restricted chromosome (GRC) of passerine birds represents an additional chromosome with a peculiar mode of non-Mendelian inheritance and tissue-specific elimination. It is usually present in two copies in female germline, while males have only a single copy, which is eliminated from the cell during spermatogenesis. The GRC shows many similarities with B chromosomes, but unlike them it seems to have some essential function for passerine birds, which prevents its loss. Using cytogenetic and genomic approaches, we analysed the GRC in several closely related estrildid finch species of the genus *Lonchura*. We show that the GRC varies enormously in size, ranging from a tiny micro-chromosome to one of the largest macro-chromosomes in the cell. Such variation can be seen not only among recently diverged species but also within species and sometimes even between germ cells of a single individual suggesting an extraordinarily dynamic nature of the GRC likely caused by frequent gains and losses of sequences on this chromosome. In one species, we observed variation in GRC copy number among male germ cells of a single individual, likely caused by unstable mitotic inheritance of this chromosome. Using crosses between the species with differently sized GRC we confirmed the maternal inheritance of the GRC, but presence of this chromosome in a small fraction of spermatozoa suggests that occasional paternal inheritance can occur in *Lonchura* species. Using comparison of genomic sequences from testis and somatic tissues, we assembled the GRC in four *Lonchura* species. Analysis of these data will shed more light into the origin of this chromosome, mechanisms its inheritance and its essential function for passerine birds.

**Acknowledgements**

The research was funded by the Czech Science Foundation (grant 23-07287S)

### S4-O3 Chromosome 3R – the donor of B chromosomes in rye, *Secale cereale*?

#### Rolf Schlegel

##### Julius Kühn Institute Quedlinburg, Gatersleben, Germany

###### **Correspondence:** Rolf Schlegel (rolf.schlegel@t-online.de)

*BMC Proceedings* 2023, **17(20)**:S4-O5

Although it was assumed for many years that B chromosomes can not chiasmatically pair with the A chromosomes of the basic genome, a first such case was described in rye. In the Japanese rye population "JNK", a plant was observed in which one of the two B chromosomes was frequently associated with one of the A chromosomes.

During metaphase I, 42% of the pollen mother cells showed this A-B chromosome pairing. The majority of associations were A-A-B trivalent. A few configurations (4.8%) were A-B rod bivalents plus an A chromosome univalent. In plants homozygous for the B chromosome even heteromorphic B-A-A-B quadrivalents were formed. Studies on late prophase cells (diplotene, diakinesis) revealed that the A-B pairings never included the nucleolus-organizer chromosome 1R. However, Giemsa staining showed that the standard B chromosome has a weak terminal C-band on the long arm, while the larger of the B chromosomes has a heavy terminal band corresponding to a chromosome arm of bilaterally banded rye chromosomes, i.e., 2R, 3R, 4R, or 7R.

When the somatic chromosomes were then examined, it turned out that in addition to the full complement of A chromosomes, there was a heteromorphic pair of B chromosomes – a standard B chromosome and a much larger second one. These two types of B chromosomes were available each homozygous or in a heterozygous constitution within the plant population. The data clearly indicated that the larger of the two B chromosomes (B’) was a non-reciprocally interchanged chromosome of A and B chromosomes. There was no A chromosome with B-segments showing an abnormal MI chromosome pairing. Obviously, a deletion of an essential part of the A chromosome was not tolerated and, therefore, eliminated from the population. The somatic and meiotic transmission of the B’ chromosome was quite regular. The gene complex causing nondisjunction seems to be present on B’, proximal to the translocation breakpoint.

A translocation between different B chromosomes could be also excluded, since pairing during meiosis ever concerned A and B chromosomes.

Apart from the few cases of artificially induced B-A translocations, this was the first case in rye where A and B chromosomes spontaneously exchanged larger chromatin segments, which led to significantly altered meiotic pairing configurations. Perhaps it was even the result of rare events of homoeologous recombination!

Using the complete set of rye primary trisomics as well as monotelo-disomics, the test crosses showed that chromosome 3R associated with B', namely the long arm of 3R.

The BS.BL-3RL translocation points to a homologous and/or homoeologous region of the standard B and chromosome 3R. Since the long chromosome arms are exchanged in both chromosomes, this could be taken as another fact for a partial structural match. A prerequisite for homologous chromosome pairing is the homologous alignment during zygotene and the formation of the synaptonemal complex.

Indeed chromosome 3R is the smallest of the genome and belongs to the ancient rye chromosomes with almost no interchanges during its ~1.7 million years evolution. DNA sequencing of flow-sorted A and B chromosomes demonstrated that the B chromosomes are rich in gene-derived sequences, which particularly corresponded to fragments chromosomes 3R (and 7R).

### S4-O4 Rapid change in genetic content of the passerine GRC

#### Stephen A Schlebusch^1^, Jakub Rídl^1,2^, Manon Poignet^1^, Francisco J Ruiz-Ruano^3,4,5,6^, Jiří Reif^7,8^, Petr Pajer^9^, Jan Pačes^2^, Tomáš Albrecht^1,10^, Alexander Suh^3,4,6^, Radka Reifová^1^

##### ^1^Department of Zoology, Faculty of Science, Charles University, Prague, Czech Republic; ^2^Institute of Molecular Genetics, Czech Academy of Sciences, Prague, Czech Republic; ^3^School of Biological Sciences, University of East Anglia, Norwich, UK; ^4^Department of Organismal Biology – Systematic Biology, Evolutionary Biology Centre, Science for Life Laboratory, Uppsala University, Uppsala, Sweden; ^5^Institute of Evolutionary Biology and Ecology, University of Bonn, Bonn, Germany; ^6^Centre for Molecular Biodiversity Research, Leibniz Institute for the Analysis of Biodiversity Change, Bonn, Germany; ^7^Institute for Environmental Studies, Faculty of Science, Charles University, Prague, Czech Republic; ^8^Department of Zoology, Faculty of Science, Palacky University, Olomouc, Czech Republic; ^9^Military Health Institute, Military Medical Agency, Prague, Czech Republic; ^10^Institute of Vertebrate Biology, Czech Academy of Sciences, Brno, Czech Republic

###### **Correspondence:** Stephen A Schlebusch (stephen.schlebusch@gmail.com)

*BMC Proceedings* 2023, **17(20)**:S4-O6

**Background**

The passerine germline-restricted chromosome (GRC) represents a taxonomically widespread example of programmed DNA elimination. This chromosome’s apparent ubiquity in the order suggests that it is indispensable, but we still know very little about the GRC’s genetic composition, function, and evolutionary significance.

**Materials and Methods**

We sequenced the testis (with the GRC) and kidney (without the GRC) of the closely related common and thrush nightingale and compared the sequencing libraries to identify GRC derived reads. These reads were used to assemble the two GRC.

**Results**

In total we identify 192 genes across the two GRC, with many of them present in multiple copies and often appearing as pseudogenized fragments. Interestingly, the genetic content of the GRC differs dramatically between the two species, despite only 1.8 million years of species divergence. Only one gene, cpeb1, has a complete coding region in all examined individuals of the two species and shows no copy number variation. The acquisition of this gene by the GRC corresponds with the earliest estimates of the GRC origin.

**Conclusions**

The GRC appears to be under little selective pressure, with rapid changes in genetic content being observed and many genes potentially being non-functional pseudogenes fragments. The standout nature of cpeb1, a gene known to play a function during oocyte maturation and early embryonic development, make it a good candidate for the functional indispensability of the passerine GRC.

**Acknowledgements**

This work is funded by the Czech Science Foundation (grant 23-07287S).

